# Aortic stent graft placement under extracorporeal membrane oxygenation in severe multiple trauma

**DOI:** 10.1002/ccr3.1127

**Published:** 2017-08-17

**Authors:** Mathias Stroehle, Wolfgang Lederer, Stefan Schmid, Bernhard Glodny, Andreas P. Chemelli, Franz J. Wiedermann

**Affiliations:** ^1^ Department of General and Surgical Critical Care Medicine Medical University of Innsbruck Innsbruck Austria; ^2^ Department of Anesthesiology and Critical Care Medicine Medical University of Innsbruck Innsbruck Austria; ^3^ Department of Radiology Medical University of Innsbruck Innsbruck Austria; ^4^ Department of Radiology Landesklinikum Baden‐Mödling Baden‐Mödling Austria

**Keywords:** Aortic dissection, extracorporeal membrane oxygenation, severe blunt trauma, trauma surgery, traumatic brain injury

## Abstract

Placement of an aortic stent graft under extracorporeal membrane oxygenation was the life‐saving procedure in a case of severe head trauma and traumatic aortic dissection after injured by a railroad engine. Timely access to neurosurgery, heart surgery, and radiology providing minimal invasive interventions increase the chances of a favorable outcome.

## Introduction

Blunt thoracic trauma causing aortic dissection is associated with high mortality exceeding 85% 1, 2, 3. Mortality is even higher in patients with additional multiple injuries. In these cases, deterioration of vital functions may progress rapidly and most victims die before hospitalization 4, 5. Patients with traumatic dissection of the thoracic aorta might need immediate vessel repair especially when circulatory failure is present 6, 7. Thoracic endovascular aortic repair (TEVAR) may be more tolerable than invasive procedures 8, 9, 10. In prolonged respiratory and circulatory failure, extracorporeal membrane oxygenation may be the last resort to maintain oxygenation. Here, we share our experience regarding treatment of a patient who underwent both extracorporeal membrane oxygenation and aortic stent graft placement during initial care.

## Case History

**Figure 1:**
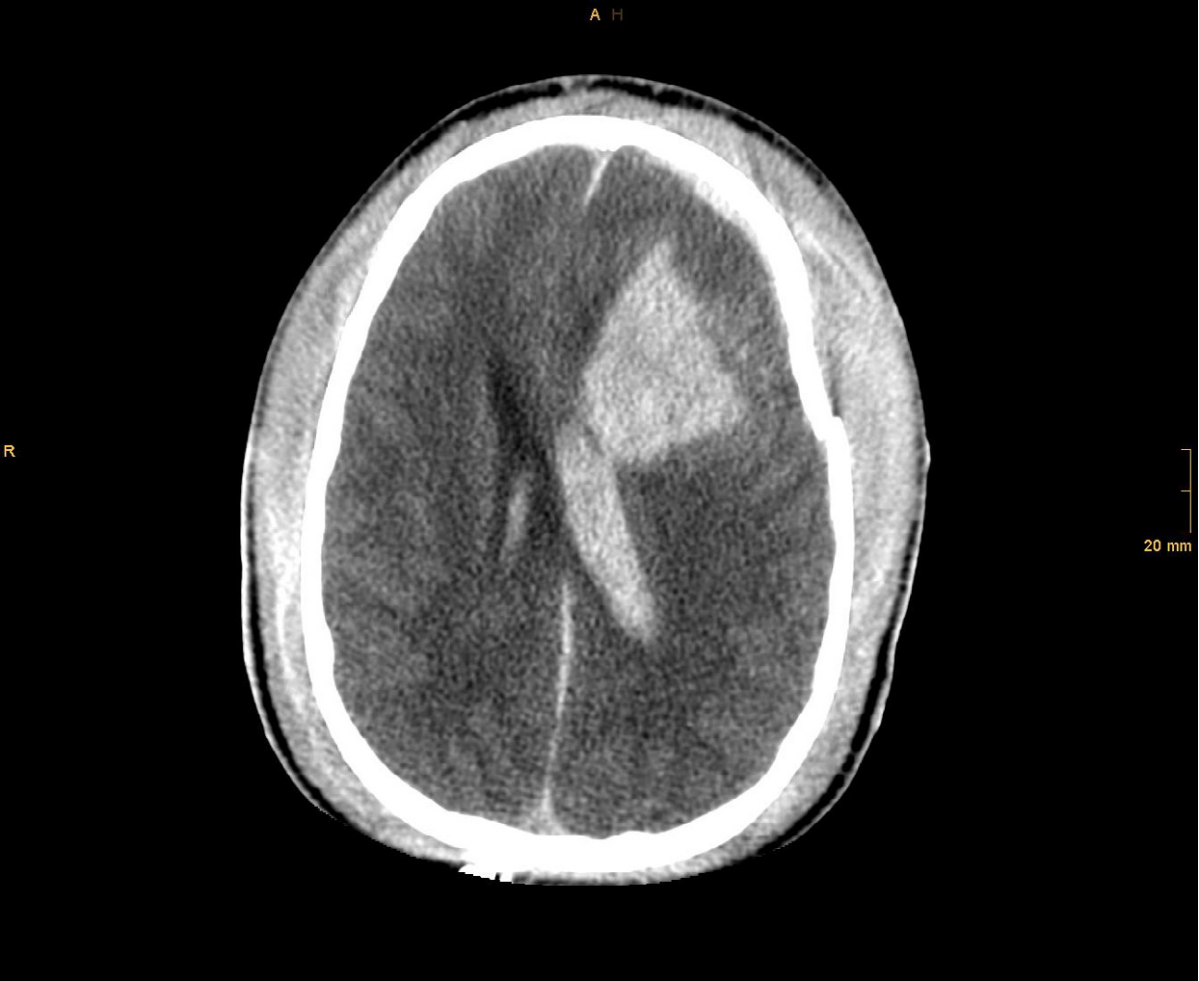
Severe traumatic brain injury with skull impression, intraventricular bleeding, midline shift, brain edema, and intracerebral bleeding.

**Figure 2:**
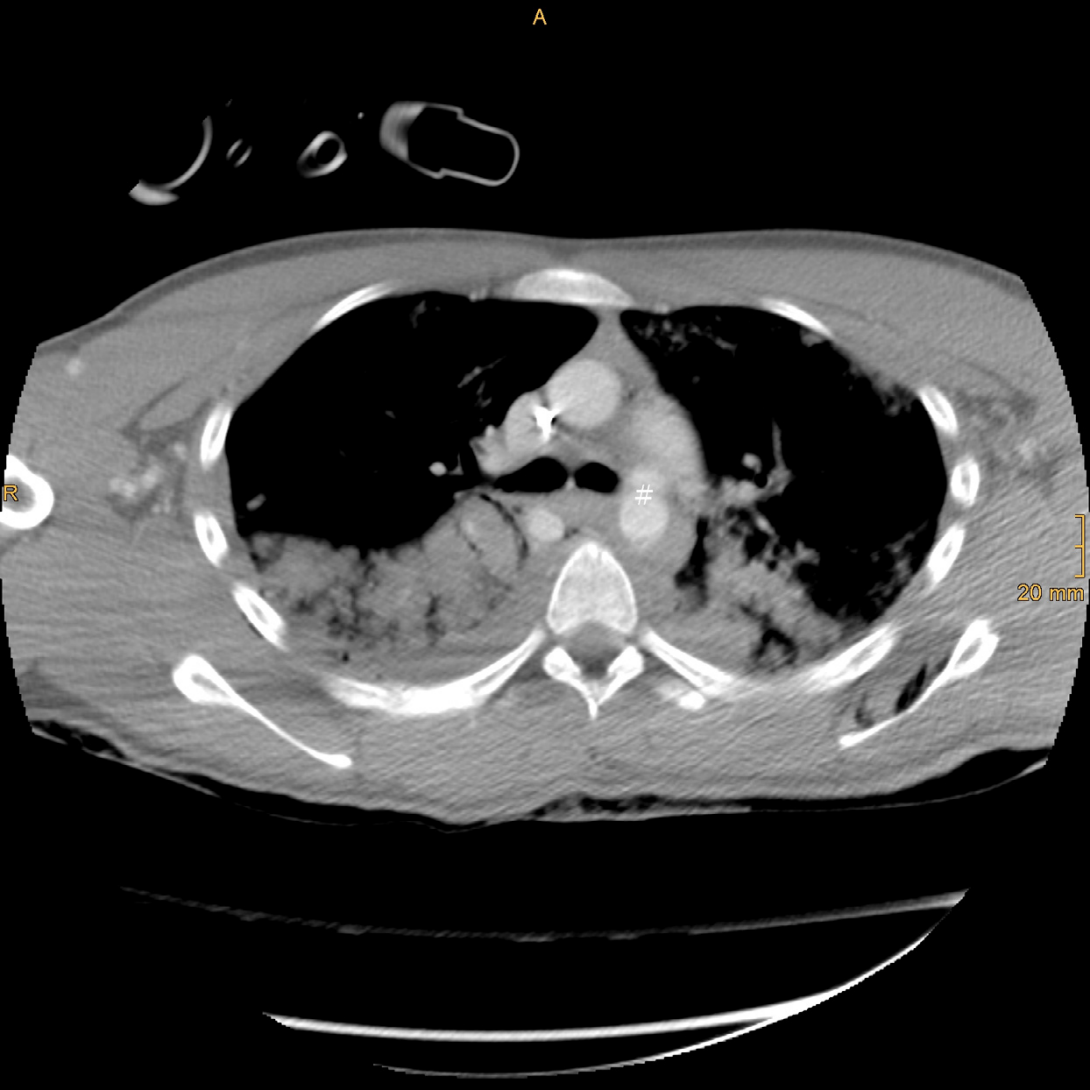
Multiple injuries in thoracic trauma; # aortic dissection, in loco typico.

A 17‐year‐old male got accidentally hit and run over by a railroad engine in the railway station during night time. When emergency medical services arrived on the scene, the patient was still under the engine. He was unconscious (GCS: 3), with bleeding from the right auditory canal and fixed left pupil. Emergency monitoring revealed severe circulatory (BP: 80/40, HR: 90/min) and respiratory failure (RR: 15, spO_2_: <80%). Following rescue, 100 mg ketamine HCl and 40 mg rocuronium bromide were administered, and tracheal intubation was performed rapidly. Mechanical ventilation mode was achieved by airway pressure release ventilation (APRV) with maximal inspiratory oxygen flow (FiO_2_: 1.0). One thousand and five hundred millilitres of crystalloid fluids and colloids was administered over three large diameter venules. In addition, blood pressure was maintained with intermittent boluses of phenylephrine and epinephrine. Within 29 min after the accident, the patient was admitted to the emergency room of a level I trauma center. Arterial blood gas examination on admission was pH 6.92, paO_2_ 29.8 mmHg, pCO_2_ 72.7 mmHg, BE ‐17 mmol/L, Horovitz oxygenation index (paO_2_/FiO_2_): 30, hemoglobin 36 g/L, glucose 18.6 mmol/L, lactate 5.4 mmol/L. Clotting parameters on admission were as follows: prothrombin time: 20%, activated partial thromboplastin time: 192 sec, fibrinogen: 89 mg/dL, platelets: 27 g/L. CT scan revealed severe injuries to the brain including left temporal open impression fracture and open otobasal fracture on the right side, multiple parenchymal hematomae, right frontal subdural hematoma, intracerebral hematoma close to the tentorium and intraventricular hemorrhage, and incipient brain edema with midline shift (Fig. 1). Thoracic injuries comprised of multiple fractures of the spine (thoracic vertebrae VII/VIII), fracture of the sternum and bilateral multiple rib fractures as well as fractures of both scapulae. Traumatic dissection of the thoracic aorta was found in the typical location (Fig. 2). Massive bilateral lung contusions and subtotal atelectasis combined with right pneumothorax and left tension pneumothorax caused profound respiratory failure. Immediate bilaterally established chest tubes did not improve oxygenation. Despite ventilation with FiO_2_: 1.0, oxygen saturation was still 79%. There was continuing severe blood loss from the nasopharyngeal region and bleeding into the chest. Blood transfusions (8 units of red blood cells, nine fresh frozen plasma) and clotting factors (prothrombin 1000 I.U., fibrinogen 6 g) were administered. Solid nasopharyngeal hemorrhage did not cease until a nasal tamponade was placed by the ENT surgeon. High‐dose catecholamines (norepinephrine (10 mg/50 mL): 20 mL/h, epinephrine (5 mg/50 mL): 5 mL/h, pitressin (100/50): 2 mL/h) kept the mean arterial pressure (MAP) above 50 mmHg minimum. Sedation was maintained with midazolam (250 mg/50 mL): 2 mL/h; analgesia was achieved with sufentanil (2.5 mg/50 mL): 2 mL/h.

**Figure 3:**
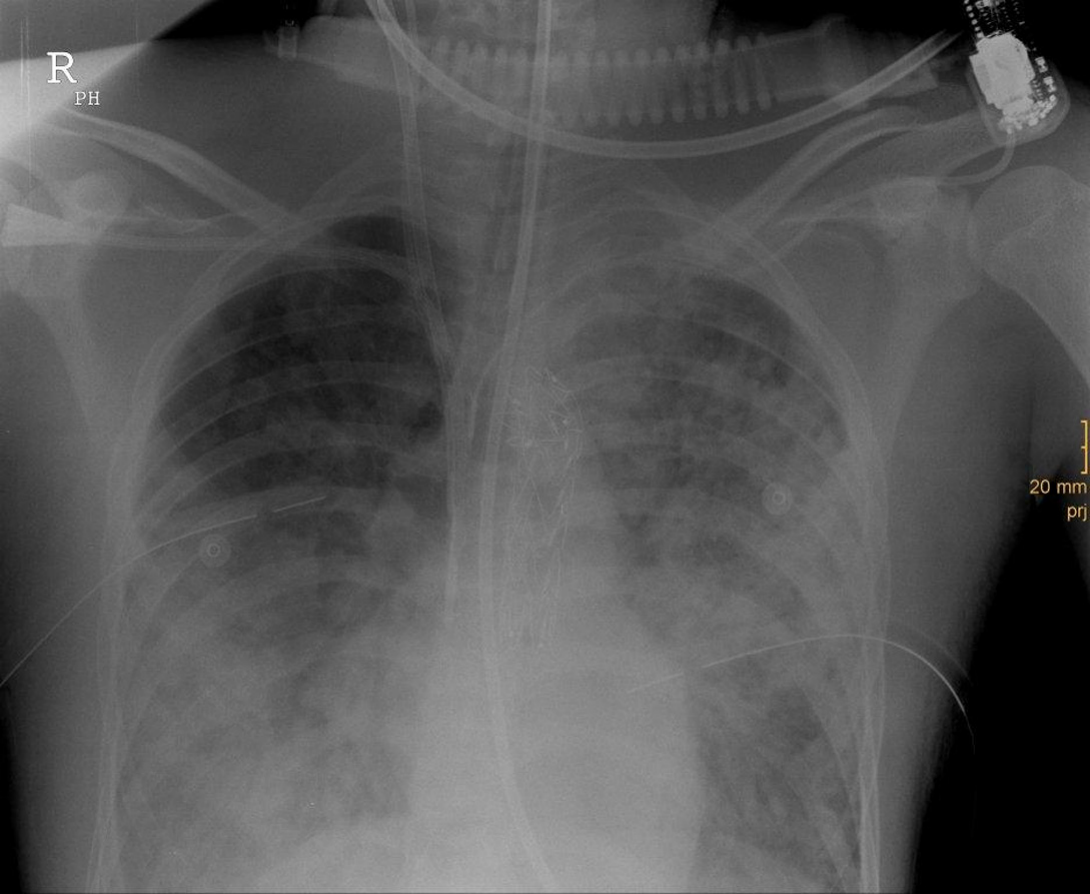
Chest X‐ray showing ARDS, bilateral chest drains, central venous lines as well as ECMO cannulas and the final position of the aortic stent graft (Valiant Thoracic, Proximal FreeFlo Straight, 24 × 127; Medtronic).

Three hours later, the patient was transferred to the ICU with an ISS score of 59. Values of arterial blood gas examination were still alarming: pH 7.03, paO_2_ 43.0 mmHg, pCO_2_ 70.0 mmHg, BE ‐10.3 mmol/L. Horovitz oxygenation index (paO_2_/FiO_2_) was 43. Due to acute respiratory distress syndrome (ARDS), the respirator was adjusted to biphasic positive airway pressure with 32/15 mbar, inversed ratio ventilation with I:E 0.6 and FiO_2_: 1.0. The neurosurgeon placed an intraparenchymatous catheter for intracranial pressure monitoring. During ICU stay, the highest intracerebral pressure (ICP) values did not exceed 35 mmHg. Cerebral perfusion pressure (CPP) was maintained keeping MAP (measured at the level of the foramen of Monro) at 60 mmHg above measured ICP.

Meanwhile, ongoing respiratory failure led to the decision to establish veno‐venous extracorporeal membrane oxygenation ((VV)ECMO) by the cardiosurgeon. Using jugulo‐femoral two‐cannula technique, a circuit was established that enabled drainage of deoxygenated blood from the right internal jugular vein and return of oxygenated blood via the left femoral vein. Flow for (VV)ECMO was maintained at 4–5 L/min. Heparinization with 5000 I.U. was discontinued due to concurrent cerebral hemorrhage and continued coagulation impairment (prothrombin time: 33%, activated partial thromboplastin time: 59 sec, fibrinogen: 140 mg/dL, D‐Dimer: 6500 *μ*g/L, platelets: 235 g/L, clotting time (InTEM) 223 sec).

Veno‐venous extracorporeal membrane oxygenation resulted in immediate improvement of patient's circulatory and respiratory condition. Arterial blood gas examination showed the following: pH 7.19, paO2 81.5 mmHg, pCO_2_ 45.3 mmHg, BE ‐11.4 mmol/L. Catecholamine requirements declined, and pitressin was discontinued. However, there was still leakage from the rupture due to dissection, and the overall condition of the patient did not support the idea of carrying out conventional thoracotomy. The radiologist performed TEVAR. Access was via the right femoral artery and the right brachial artery in supine position. During positioning and deployment of the stent graft (Valiant Thoracic, Proximal FreeFlo Straight, 24 × 127; Medtronic, Fridley, MN, USA), Fig. 3 the systolic blood pressure had to be reduced and maintained at 90 mmHg for a few minutes. Otherwise, MAP was kept between 90 and 110 mmHg. The completion angiogram displayed sufficient sealing of the rupture site. Again, aggregation/antiaggregant therapy was an issue of discussion, but heparinization was not initiated by continuous perfusion (5000 I.U./50 mL) until the next day keeping activated clotting time above 120 sec. Antibiotic treatment was started with cefuroxime and then switched to combined ceftriaxone and fosfomycin.

Veno‐venous extracorporeal membrane oxygenation was discontinued on day six when FiO2: 0.5 and APRV ventilation provided adequate gas exchange and circulation was sufficiently supported with moderate doses of norepinephrine. However, safe recovery was still far away and the postoperative period remained eventful. Disassembly of (VV)ECMO was followed by partial ischemia of the left lower limb and thrombosis of iliac and inferior cava vein necessitating infrarenal placement of a cava filter. Concurrently, cerebral edema was increasing as was a right frontoparietal hygroma. Vasospasm of cerebral arteries was treated with nimodipine.

Weaning from respirator turned out to be difficult and extubation was not possible without thoracotomy to remove intrapleural hematoma. Forty‐two days after admission to the ICU, the patient was transferred to the neurologic ward. Brainstem auditory evoked potentials with series of clicks to each ear indicated peripheral dysfunction. Sensory evoked potentials administered to the lower limbs revealed slowing of electrical conduction in the left tibial nerve. Neuropsychological investigation 4 months after the accident demonstrated lasting psychomotor retardation and impaired memory.

Despite intense and prolonged rehabilitation, the patient did not regain ability to work, but was able to live at home without needing high degree of care.

## Discussion

Combined cerebral and thoracic trauma in patients with multiple trauma show the limits of modern intensive care. Hypovolemia and hypoxemia are detrimental to the brain. Increasing MAP to maintain adequate CPP by administration of volume, blood transfusions and catecholamines increases blood loss and clotting disorders unless surgical hemostasis can be achieved. In our case, the episode of hypotension was <45 min after the accident, but prolonged hypoxemia lasted until (VV)ECMO. It is impossible to say with any certainty whether prolonged ischemia/hypoxia had an impact on the neurologic sequelae in our patient or whether they resulted entirely from direct brain injury. As soon as intracranial pressure monitoring was achieved, CPP was maintained between 50 and 70 mmHg in our patient. Both low and excessive CPP have been reported to be associated with poor outcome 11.

Persistent hypoxemia and hypercarbia in the initial phase necessitated the use of (VV)ECMO. Cannulas were placed in the right jugular vein and left femoral vein to allow access for additional angiographic interventions. The procedure was carried out under ultrasound guidance as in severe hypoxia, arterial blood can resemble venous blood 12. Raising MAP to maintain CPP above 60 mmHg increased the risk of aggravating the untreated aortic dissection and rupture. Surgical treatment of the impaired aorta using conventional thoracotomy with cardiopulmonary bypass and high‐dose heparin was no option.

Thus, minimal invasive TEVAR via right femoral artery and right brachial artery appeared to be the least hazardous intervention 13. Although not ideal, antiaggregant therapy according to protocol was not carried out in the first 4 days because of solid nasopharyngeal bleeding and intracerebral and pulmonary hemorrhage. Unfortunately, delayed heparinization was associated with thrombosis of iliac and inferior cava vein.

There are only a few published reports on the successful placement of a TEVAR under the conditions of ECMO for life‐threatening circulatory and respiratory failure. Severe multiple trauma patients with combined brain injuries and ARDS represent a challenge to intensive care. Sound interdisciplinary cooperation between different specialties is of paramount importance. Rapid emergency response, short period of out‐of‐hospital treatment, rapid transport to a major trauma center with timely access to surgical and medical facilities including neurosurgery, heart and thoracic surgery, and radiology providing minimal invasive interventions increase the chances of a favorable outcome.

## Authorship

MS: helped to collect patient documents, made the literature research, and wrote the manuscript. WL: made the literature research and wrote the manuscript. SS: reviewed the draft and wrote the critical care part. BG: collected the pictures and helped to write the manuscript. APC: treated the patient and helped to write the manuscript. FJW: treated the patient, collected patient documents, designed the manuscript, and wrote the manuscript.

## Conflict of Interest

None declared.
